# The Treatment of Breast Cancer Using Liposome Technology

**DOI:** 10.1155/2012/212965

**Published:** 2012-02-21

**Authors:** Sarah Brown, David R. Khan

**Affiliations:** Department of Mathematics, Chemistry and Physics, West Texas A&M University, Canyon, TX 79016-0001, USA

## Abstract

Liposome-based chemotherapeutics used in the treatment of breast cancer can in principle enhance the therapeutic index of otherwise unencapsulated anticancer drugs. This is partially attributed to the fact that encapsulation of cytotoxic agents within liposomes allows for increased concentrations of the drug to be delivered to the tumor site. In addition, the presence of the phospholipid bilayer prevents the encapsulated active form of the drug from being broken down in the body prior to reaching tumor tissue and also serves to minimize exposure of the drug to healthy sensitive tissue. While clinically approved liposome-based chemotherapeutics such as Doxil have proven to be quite effective in the treatment of breast cancer, significant challenges remain involving poor drug transfer between the liposome and cancerous cells. In this review, we discuss the recent advancements made in the development of liposome-based chemotherapeutics with respect to improved drug transfer for use in breast cancer therapy.

## 1. Introduction

A significant challenge in the treatment of cancer involving chemotherapy is the efficient delivery of cytotoxic agents to tumor tissue while at the same time minimizing the undesired negative side effects associated with these drugs. The use of drug delivery systems (DDSs) such as liposomes can alter drug pharmacokinetics and biodistribution in a manner that improves the overall pharmacological properties of commonly used chemotherapeutics. Liposomes are particularly attractive DDS in part due to the ease with which they can be generated and modified such that they can be used to treat a wide variety of cancers [1–3]. Breast cancer in particular has been the focus of many studies involving liposome-based chemotherapeutics in part due to the clinical success of various drugs such as Doxil, which is a liposomal formulation currently used to treat recurrent breast cancer [4–6]. Doxil is a liposomal preparation composed of the relatively high phase-transition temperature phospholipid hydrogenated soy phosphatidylcholine (HSPC) and cholesterol [7, 8] resulting in a stable DDS with enhanced bilayer rigidity. The anthracycline doxorubicin is the active cytotoxic agent and is contained within the internal aqueous core of the liposome. The encapsulation of doxorubicin within liposomes significantly decreases the cardiotoxicity that commonly results from the use of unencapsulated anthracyclines by decreasing the amount of the drug being delivered to the heart [9, 10]. Thus, patients can receive much higher doses of the chemotherapeutic in the liposomal formulation compared to unencapsulated, thereby allowing tumor tissue to potentially be exposed to a lethal dose of the drug while minimizing deleterious side effects. This inherent advantage associated with the use of liposomes as drug delivery vehicles also serves to minimize the many other toxic side effects associated with doxorubicin to include gastrointestinal toxicity and complications arising from myelosuppression [10, 11]. However, while liposome-based drugs such as Doxil have proven to be effective, significant challenges remain involving future improved formulations, particularly with respect to drug transfer between the DDS and cancerous cells. This review discusses the benefits and challenges associated with the use of liposome-based chemotherapeutics in the treatment of breast cancer and also addresses the recent advances made in the field with respect to improved formulations aimed to surmount some of these obstacles. Amongst the strategies discussed here, we discuss designs intended to improve drug release within the tumor microenvironment and/or colocalization between the drug and breast cancer cells to include temperature-sensitive liposomes and targeted liposomes. 

## 2. Delivery of Pegylated Liposome-Encapsulated Drugs

 Liposomes have long been recognized as drug delivery vehicles for chemotherapeutics since they were first described in the 1960s. They are well suited for this purpose as they can accommodate both hydrophilic and hydrophobic drugs by storing them either in their internal aqueous core or their phospholipid bilayer, respectively. The mere fact that liposomes are generated from phospholipids makes them ideal candidates for drug delivery systems as they are nontoxic and biodegradable. In addition to being biocompatible, the phospholipid bilayer prevents the encapsulated active form of the drug from being broken down in the body prior to reaching tumor tissue and also minimizes exposure of the encapsulated drug to healthy tissue while in circulation. Both of these effects serve to increase the therapeutic indices of drugs as elevated levels of the active form of the drug is delivered to the tumor site such that the intended cytotoxic effect is achieved, while at the same time unintended negative side effects of the drug are substantially reduced when compared to the unencapsulated form. For example, while proving to be quite efficient when used in clinical settings to treat various types of cancers, liposomal treatment has been shown to dramatically reduce some of the traditional side effects associated with chemotherapy, such as nausea and vomiting when compared to unencapsulated drugs [12]. 

 An important physical aspect associated with the clinical successes of liposome-based drugs is the overall size of the nanocarrier. While the size of these drug delivery systems can be carefully controlled, liposomes intended for the delivery of chemotherapeutics tend to be ~50–100 nm in diameter. This lower-size limit prevents these predominately intravenous based drugs from randomly penetrating normal vessel walls while in circulation. As far as the upper size limit, it may appear as if larger systems would be ideal based on the fact that more of the cytotoxic agent could potentially be delivered to the tumor site; however, there is an upper size limit to these systems. In order to gain access to tumor tissue, it is imperative that these drugs retain the ability to extravasate from circulation through the large vascular defects known to be present in and around tumor sites attributed to constant ongoing angiogenesis previously reported to be ~250 nm or greater [13]. Therefore, liposome-based chemotherapeutics whose overall size is below this threshold have the potential to accumulate within tumor tissue based on this form of “passive” drug delivery. This coupled with the fact that drug retention within the tumor is generally high attributed to the poor lymphatic drainage observed within tumors results in a phenomenon known as the enhanced permeation and retention (EPR) effect [14–16]. Another major limiting factor with respect to the size of these drug delivery systems relates to circulation times *in vivo*. The general trend for liposomes of similar phospholipid compositions is that increasing size results in escalating uptake by the reticuloendothelial system (RES) [17]. In fact, previous studies have shown that liposomes 250 nm in diameter are removed more than twice as fast from circulation when compared to liposomes 100 nm in diameter of similar phospholipid compositions [18]. This is particularly problematic as it is imperative that these systems remain in circulation long enough such that they can accumulate within tumor tissue at levels great enough to have the intended cytotoxic effect. One obvious method for overcoming this obstacle involves the overall size reduction of the nanocarrier, which as mentioned earlier also has the unfortunate effect of translating into less drug being delivered by the nanocarrier. Another proven method for overcoming this obstacle without compromising the amount of chemotherapeutic being delivered to tumors is the surface coating of these drug delivery vehicles with polymers, particularly polyethylene glycol (PEG). This generates “Stealth” liposomes, which is a name given to them based on their ability to evade the immune system resulting in significant increases in circulations times *in vivo* [14, 19, 20]. In fact, the benefit of pegylation is quite apparent when comparing the relative half-lives of nonpegylated and pegylated liposomes which increases from just a few hours to as much as 45 hours, respectively [9]. Therefore, it is not surprising to note that the clinically approved drug Doxil is in fact pegylated (M_r_ 2000) in order to improve tumor site accumulation of the drug [14]. However, while surface coating liposomes with PEG achieve desirable circulation times *in vivo*, it also negatively influences tumor cellular uptake of these systems as the presence of the PEG moiety presents a steric barrier between the drug and cancer cells [10]. Therefore, while pegylation does not eliminate cellular uptake entirely, delivery of pegylated liposome-based chemotherapeutics is in large part based on the ability of the encapsulated drug to escape or be released from the nanocarrier via leakage in the tumor microenvironment prior to tumor cellular uptake of the free drug. Therefore, future strategies involving the improved delivery efficiency of pegylated liposome-based drugs, particularly in the treatment of breast cancer, are aimed at various enhanced triggered release techniques to facilitate this process. One such method involves the heat-triggered release of pegylated thermosensitive liposomes. 

### 2.1. Hyperthermia and Improved Liposome-Based Drug Delivery

**Figure 1:**
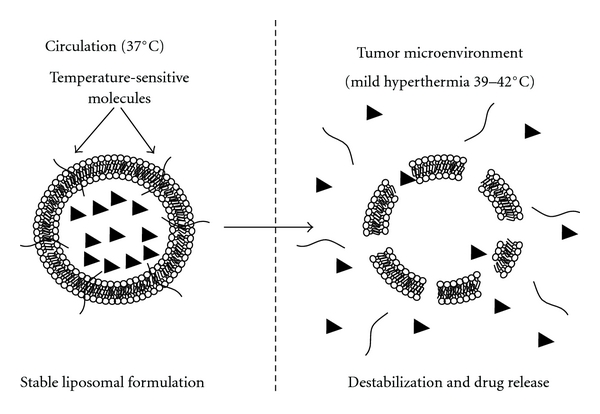
Temperature-sensitive liposomes designed to remain stable while in circulation at 37°C and become significantly destabilized in the tumor microenvironment at slightly higher temperatures 39–42°C.

 While liposome-based drugs of the appropriate size retain the ability to extravasate out of circulation at tumor sites, various challenges remain involving release of the encapsulated drug from the nanocarrier. Therefore, one aspect with respect to the future design of these drugs involves the incorporation of various molecules within liposomal formulations that respond to external stimuli in a manner that disrupts liposomes to allow for the delivery of encapsulated material. While there have been many methods reported recently aimed to accomplish this in order to treat a wide variety of cancers, thermosensitive molecules added to these formulations specifically for the purposes of treating breast cancer have proven to be quite effective. These temperature-sensitive liposomes are designed to be stable at the normal physiological temperature of 37°C but become significantly destabilized at slightly higher temperatures (Figure 1). The use of liposomes as the nanocarrier in these formulations is a particularly attractive option with respect to both enhanced tumor site accumulation, as well as facilitated release of the encapsulated drug. This is attributed to the fact that a local increase in temperature has been shown to enhance extravasation of liposomes out of circulation resulting in their preferential accumulation to the heated tumor [21], and that liposomes are known to become destabilized at elevated temperatures [1, 2]. For example, we and others have previously shown that liposomes composed of various phospholipids are much leakier at 37°C than those stored at 4°C [1, 3, 22]. Thus, the use of temperature-sensitive liposomes to deliver encapsulated chemotherapeutics to solid tumors such as breast cancer is an area of promising research, and many successful constructs have previously been reported. For example, liposomes composed of dipalmitoylphosphatidylcholine (DPPC), monostearoylphosphatidylcholine (MSPC), and distearoylphosphatidylethanolamine (DSPE)-PEG 2000 are currently in Phase II clinical trials for the treatment of recurrent breast cancer (http://www.celsion.com). These lyso-lipid temperature-sensitive liposomes encapsulate doxorubicin and have previously been shown to exhibit enhanced drug release rates under mild hyperthermic conditions while remaining relatively stable at normal physiological temperature [23]. More recently, Tagami et al. have reported a similar liposome-based system in which the minor component MSPC is replaced with a nonionic surfactant Brij78 [24]. This new formulation outperformed the lyso-lipid temperature-sensitive liposomes when tested in mice inoculated with a mammary carcinoma cell line (EMT-6). Chen et al. have also reported promising results using thermosensitive liposomes prepared with DPPC, 1-myristoyl-2-palmitoyl phosphatidylcholine (MPPC), and DSPE-PEG 2000 [25].

### 2.2. Targeted Liposome-Based Chemotherapeutics

**Figure 2:**
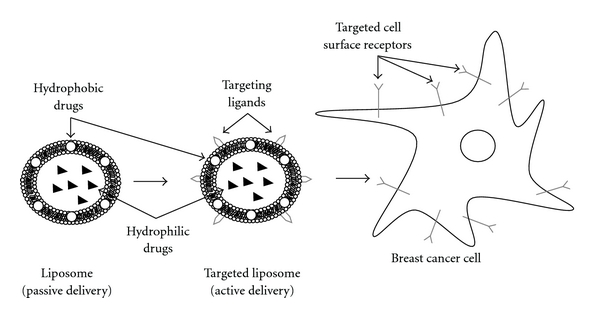
Liposomes can accommodate both hydrophobic and hydrophilic drugs either in the phospholipid bilayer or in the internal aqueous core, respectively. They can be used in passive delivery of drugs or in active delivery in which targeting ligands are added. Targeting ligands are selected based on various upregulated cell-surface receptors present on cancer cells with respect to normal cells.

**Figure 3:**
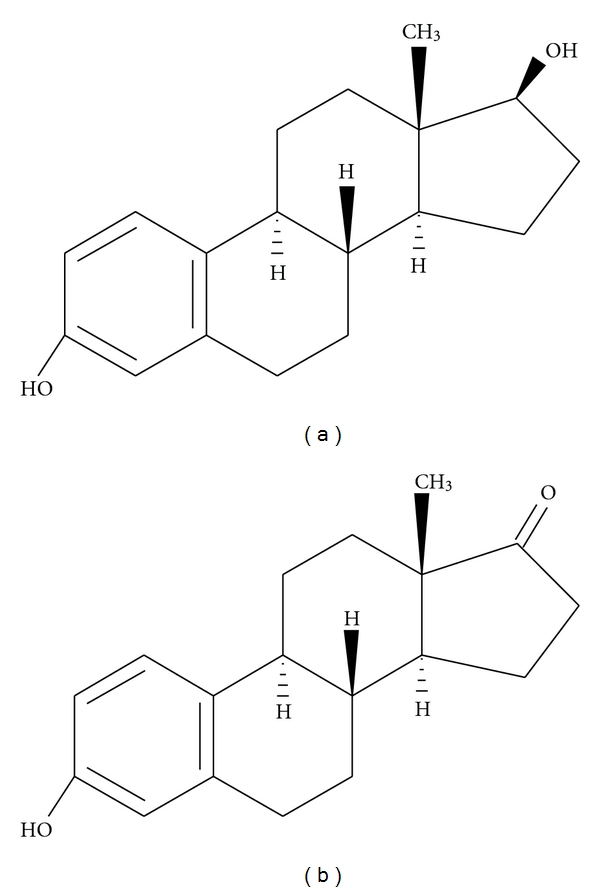
Both estradiol (a) and estrone (b) have previously been used as targeting ligands in liposome-based chemotherapeutics against breast cancer.

**Figure 4:**
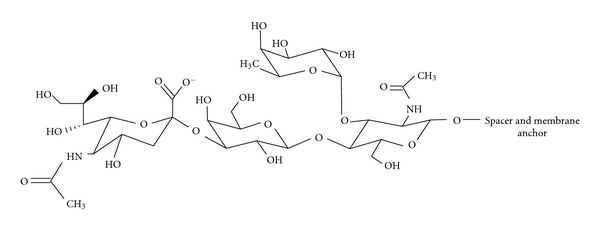
Structure of the tetrasaccharide Sialyl Lewis X used in the carbohydrate vector (which includes a spacer and membrane anchor) to target lectins known to be overexpressed by mammalian malignant cells when compared to normal.

 Another strategy employed in order to potentially increase the overall therapeutic index of liposome-based drugs involves improving the colocalization between the chemotherapeutic and breast cancer cells. In some cases, this strategy may also involve improvement of cellular internalization of the whole liposome-based drug, particularly when cell-surface receptors known to undergo receptor-mediated endocytosis is concerned. Generally, these types of formulations involve surface modifications made to liposomes in order to accommodate targeting ligands which are specific for known upregulated breast cancer cell-surface receptors (Figure 2), and several promising constructs have recently been reported (Table 1). For example, anti-HER2 immunoliposomes have been shown to be far more effective against HER2-overexpressing breast cancer cells when compared to nontargeted liposomes [26]. In this study, the targeted liposomes were formulated with Fab of recombinant humanized anti-HER2 monoclonal antibody. Immunoliposomes containing anti-transferrin receptor antibody and loaded with siRNA have been successfully used in breast cancer animal models [28]. Similarly, siRNA-loaded liposomes surface modified to contain a peptide which preferentially binds a specific breast cancer cell line have recently been shown to exhibit notable targeting capabilities [27]. A particularly attractive target with respect to breast cancer is the estrogen receptor (ER) which is overexpressed in a large number of breast cancer cells [32, 33]. For example, estradiol has previously been incorporated into liposomes for use as a targeting ligand against ER-expressing breast cancer cells [29]. More recently, Paliwal et al. have reported a targeted liposomal-based chemotherapeutic which utilizes a structurally similar molecule, estrone instead of estradiol (Figure 3) as the targeting ligand [30]. The tumor accumulation of the targeted liposomes in this latter and most recent study was approximately 6 times higher than the observed accumulation with nontargeted liposomes. Targeted liposomes have also been generated using a specific carbohydrate vector, which have been shown to have enhanced tumor growth inhibition compared to their nontargeted counterparts when tested *in vivo* in a mouse breast cancer model [31]. In this study, a SiaLe^X^ vector was used as the targeting ligand which targets lectins, specific carbohydrate-binding proteins known to be overexpressed by mammalian malignant cells when compared to normal. The vector construct was designed to essentially contain three parts for liposome incorporation to include Sialyl Lewis X (Figure 4), a spacer, as well as a membrane anchor.

## 3. Conclusions

 The major overall goal in the design of liposome-based chemotherapeutics is to generate a formulation that is stable while in circulation, yet efficiently deliver encapsulated cytotoxic agents to tumor tissue. Currently, clinically approved drugs to treat breast cancer such as Doxil are relatively stable in circulation; however, drug transfer from the nanocarrier to breast cancer cells remains particularly problematic. This is in part attributed to the fact that DDSs of this size (~100 nm in diameter) require pegylation to achieve optimal circulation times *in vivo*, which negatively influences cellular uptake of these systems. One solution to this problem involves making liposomes smaller in size. For example, other clinically approved liposome-based drugs such as DaunoXome currently used to treat Kaposi's sarcoma do not need to be pegylated as a result of their small size reported to be ~45 nm in diameter [34]. An additional advantage that smaller DDS may have over their larger counterparts also involves their ability to potentially penetrate deeper into the tumor microenvironment [35]. However, it remains controversial as such small systems are potentially limited in their ability to deliver an effective dose of the drug to tumor tissue. Thus, several groups are currently working on improved formulations that retain adequate circulation times *in vivo*, yet more efficiently deliver their encapsulated cargo without having to necessarily reduce the overall size of the nanocarrier. Many of these systems have been reported here and include formulations designed to release encapsulated cytotoxic agents at elevated temperatures and/or improve colocalization between the drug and breast cancer cells through targeting ligand addition. It is worth noting that liposomal formulations involving both targeting ligand incorporation as well as pegylation can be particularly challenging as the presence of the PEG moiety has the ability to potentially negatively influence receptor/ligand recognition [3]. Nonetheless, the systems reported here or similar formulations may in fact be commonly used clinically in the near future in order to more effectively treat breast cancer.

## Figures and Tables

**Table 1:** Recently reported targeted liposome-based chemotherapeutics to treat breast cancer. PE38KDEL from reference [26] is a 38 kDa mutant form of pseudomonas exotoxin A (PE), and the peptide sequence from reference [27] is DMPGTVLP.

Cell-surface target	Targeting ligand	Encapsulated cargo	Reference
HER2	Anti-HER2 Fab'	PE38KDEL	[26]
Transferrin receptor	Antitransferrin receptor antibody	HER-2 siRNA	[28]
MCF-7 cell specific	Peptide	PRDM14 siRNA	[27]
Estrogen receptor	17*β*-estradiol	Anticancer gene	[29]
Estrogen receptor	Estrone	Doxorubicin	[30]
Lectins	Selectin ligand (SiaLe^X^)	Merphalan	[31]
